# Continuous infusion of lipo-prostaglandin E1 for Takayasu’s arteritis with heart failure in an 11-month-old baby: a case report

**DOI:** 10.1186/s13256-018-1769-x

**Published:** 2018-09-02

**Authors:** Ryo Higaki, Aya Miyazaki, Yujiro Tajiri, Mikihito Shoji, Shun Saito, Shin-ichiro Yoshimura, Naoki Miki, Kazuhiro Hatta, Hiraku Doi

**Affiliations:** 1 Department of Pediatrics, Tenri Hospital, 200 Mishima-cho, Tenri, Nara 631-8552 Japan; 2 Department of Pediatric Cardiology, Tenri Hospital, 200 Mishima-cho, Tenri, Nara 631-8552 Japan; 3 Department of Rheumatology, Tenri Hospital, 200 Mishima-cho, Tenri, Nara 631-8552 Japan

**Keywords:** Takayasu’s arteritis, Infant, Baby, Heart failure, Abdominal vascular stenosis, Lipo-prostaglandin E1

## Abstract

**Background:**

Takayasu’s arteritis is extremely rare in children aged below 6 years. At the onset of Takayasu’s arteritis in children, symptoms are varied but differ from those in adults. Corticosteroids are the mainstay of treatment for preventing irreversible vascular damage but there is no standard treatment for progressive vascular stenosis.

**Case presentation:**

A Japanese 11-month-old baby boy presented with Takayasu’s arteritis and heart failure, possibly due to afterload mismatch caused by high blood pressure. Computed tomography was performed and revealed thoracic and abdominal aortic aneurysms. It also revealed severe celiac artery stenosis and bilateral renal artery stenosis. Prednisolone was initiated as first-line therapy. The fever resolved, and C-reactive protein levels returned to normal. Although his general condition improved, deterioration of vascular lesions was evident. Celiac artery occlusion, severe right renal artery stenosis, and new superior mesenteric artery stenosis were observed. We decided to use a continuous infusion of lipo-prostaglandin E1 for prevention of branch stenosis of his abdominal aorta. The progression of vascular stenosis was stopped and our patient’s cardiac function gradually improved.

**Conclusions:**

A differential diagnosis of heart failure with high blood pressure should be considered in babies. The progression of vascular stenosis may be suppressed by lipo-prostaglandin E1.

## Background

Takayasu’s arteritis (TA) is a chronic inflammatory disease that causes stenosis, occlusion, and aneurysm of the aorta and its major branches and then progresses to damaging major organs such as the brain, heart, and kidneys. It occurs predominantly in young women aged 20–40 years [1]. There are limited reports of the disease onset in children, particularly in babies. At the onset of TA in children, symptoms are varied but differ from those in adults. The most frequent presentation in children is hypertension (82.6%), followed by headache (31%), fever (29%), dyspnea (23%), weight loss (22%), and vomiting (20.1%) [2]. It is of the utmost importance to prevent irreversible vascular damage in these patients. Corticosteroids are the mainstay of treatment, and attempts have been made to use other immunosuppressants for second-line therapy. There is no standard treatment for progressive vascular stenosis [3, 4].

We present the case of an 11-month-old baby boy with heart failure who was given the tentative diagnosis of cardiomyopathy on admission, which was finally confirmed as TA. We used a continuous infusion of lipo-prostaglandin E1 (lipo-PGE1), a liposomal preparation of prostaglandin E1 (PGE1) in which the active ingredient is enclosed in lipid microspheres, to prevent branch stenosis of his abdominal aorta.

## Case presentation

A Japanese 11-month-old baby boy presented at our institution with symptoms including fever, weight loss, and gallop rhythm. His fever persisted for 3 days before presentation but no treatment was provided. He was born by vaginal delivery at 37 weeks of gestation with a weight of 2612 g (36th percentile) and a head circumference of 33.7 cm. There was no family history of aortic disease and sudden death. When he was 7-months old, he had a fever of unknown origin that persisted for 2 weeks. He was poor in weight gain and was 7.55 kg (6th percentile) at the age of 10 months; his body weight decreased by 0.57 kg in the 3 weeks before presentation.

**Fig. 1:**
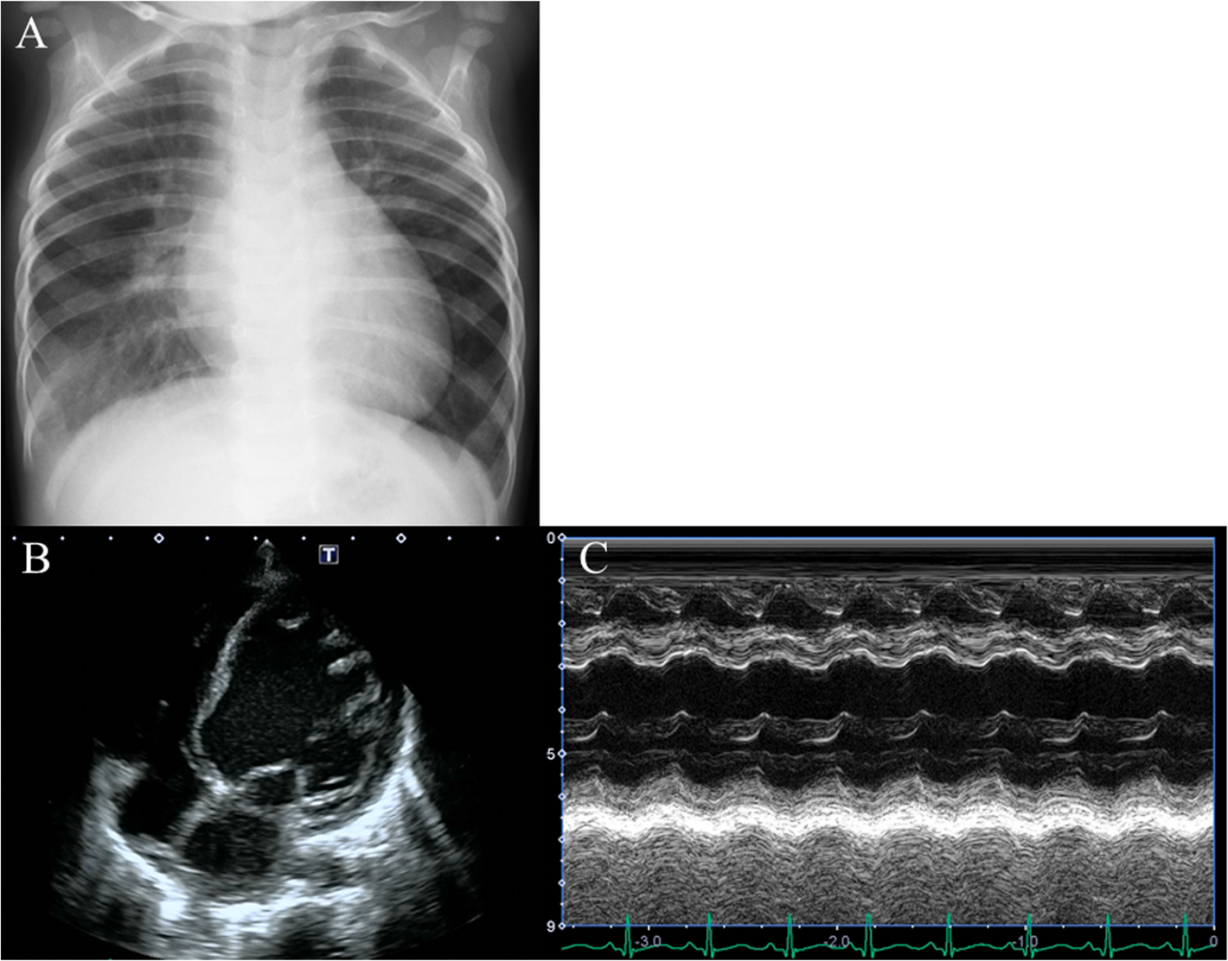
Images taken at admission. **a** Chest X-ray. **b**, **c** Four-chamber and M-mode view of echocardiography, respectively

On examination at the presentation, his height was 70.8 cm, his weight was 6.98 kg, and his head circumference was 43 cm. He was ill-appearing and febrile to 38.4 °C. His blood pressure was 124/62 mmHg and pulse 146/minute. There was a notable S3 gallop and systolic murmur at the apex (Levine scale grade III/VI); however, there were no signs of rales or peripheral edema. Other physical and neurological examinations were normal. A chest X-ray revealed cardiomegaly, with 58% cardiothoracic rate (Fig. 1a). Echocardiography indicated left ventricular (LV) enlargement and dysfunction with LV diastolic dimension of 32 mm (130% of normal), LV ejection fraction 48% (Fig. 1b, c), moderate mitral regurgitation, and slight aortic regurgitation. Blood tests indicated the following: white blood cell count, 11.07 × 10^3^/μl; hemoglobin, 10.6 g/dl; C-reactive protein, 5.59 mg/dl; creatine phosphokinase, 294 U/l; creatine phosphokinase-MB isozyme, 27 U/l; fibrin degradation products D-dimer, 2.1 μg/ml; brain natriuretic peptide, 2841 pg/ml; human atrial natriuretic peptide, 1360 pg/ml; and serum troponin T, 0.26 ng/ml. His blood culture at admission was negative.

**Fig. 2:**
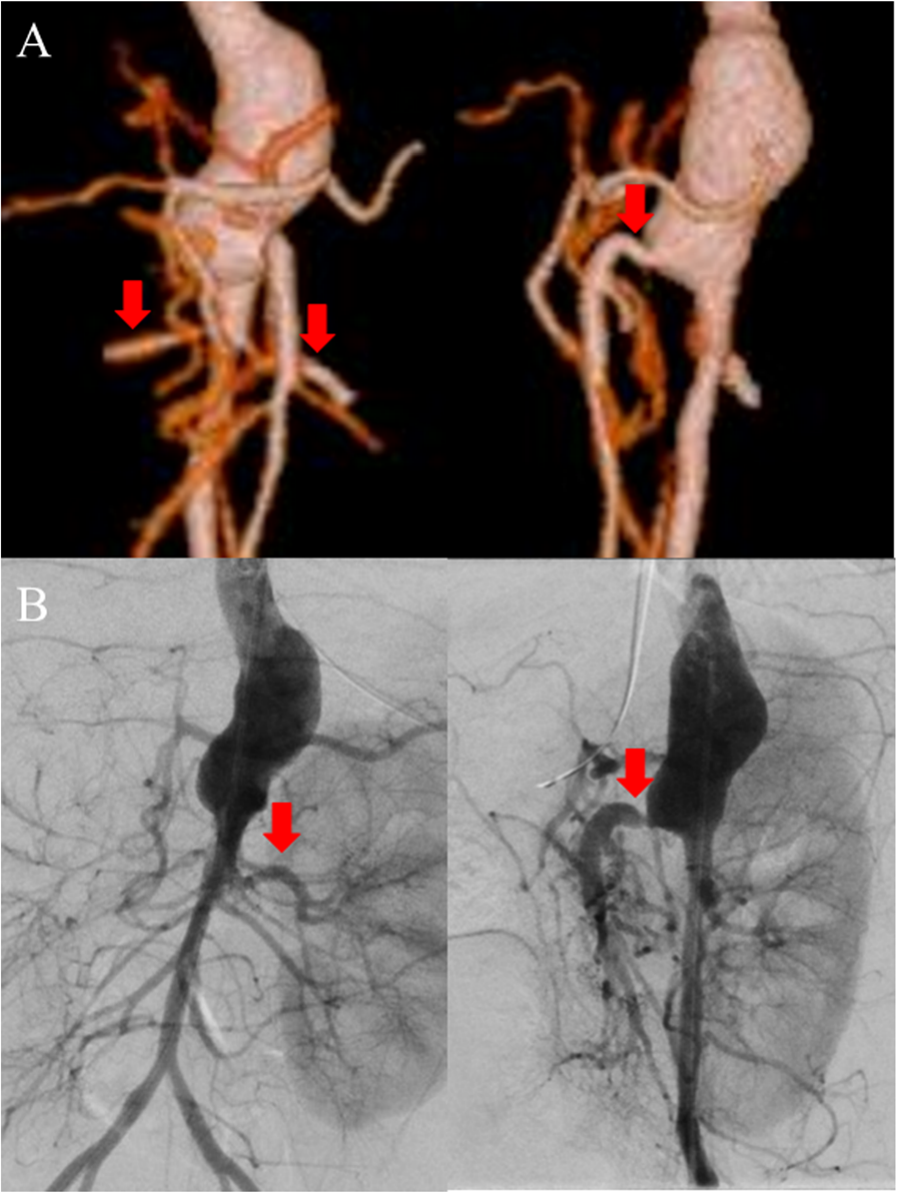
**a** Three-dimensional computed tomography on hospital day 3. Frontal (*left*) and left lateral (*right*) view; *arrows* show bilateral renal arteries (*left figure*) and superior mesenteric artery (*right figure*). **b** Angiographic image on hospital day 45. Frontal (*left*) and left lateral (*right*) view. The *arrow* shows the left renal artery (*left figure*) and superior mesenteric artery stenosis (*right figure*). Right renal artery is not contrasted

As dilated cardiomyopathy was diagnosed, we initiated diuretics. However, his fever and high blood pressure (systolic blood pressure, 130–140 mmHg), which rarely present with dilated cardiomyopathy, persisted. To investigate the causes of high blood pressure, computed tomography was performed and revealed thoracic and abdominal aortic aneurysms on hospital day 3 (Fig. 2a). It also revealed severe celiac artery stenosis and bilateral renal artery stenosis. From these findings, TA was diagnosed and 1 mg/kg per day prednisolone was consequently initiated as first-line therapy. His fever had resolved on day 5; his C-reactive protein levels returned to normal on day 10.

**Fig. 3:**
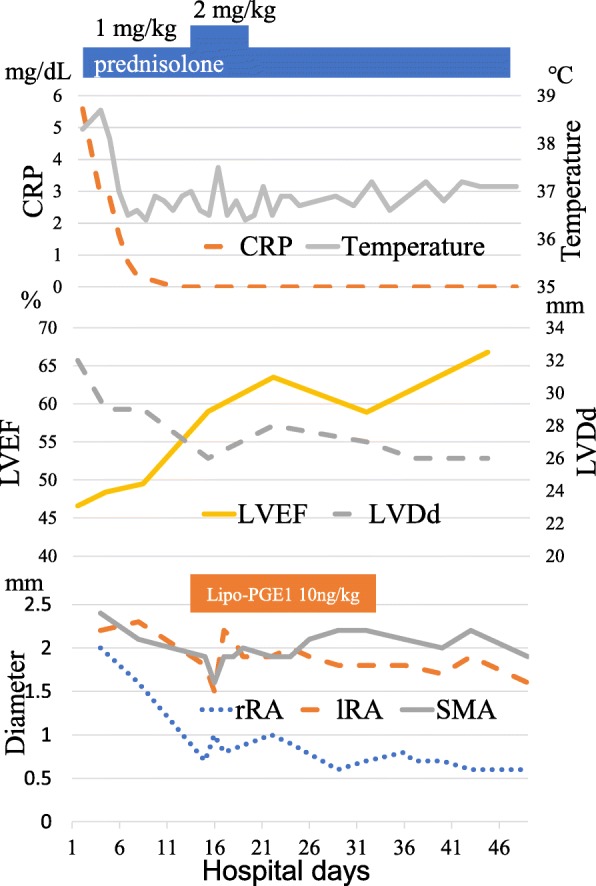
Treatments, inflammatory parameters, and the diameter of vascular branches by echocardiography. *CRP* C-reactive protein, *lipo-PGE1* lipo-prostaglandin E1, *lRA* left renal artery, *LVDd* left ventricular end-diastolic dimension, *LVEF* left ventricular ejection fraction, *rRA* right renal artery, *SMA* superior mesenteric artery

Although his general condition improved, deterioration of vascular lesions was evident, as shown by echocardiography, on day 15. Celiac artery occlusion, severe right renal artery stenosis, and new superior mesenteric artery stenosis were also observed on day 15. We increased the dose of prednisolone to 2 mg/kg per day for 1 week due to the possibility of active inflammation around vascular lesions. In addition, we attempted continuous intravenous infusion of lipo-PGE1 at 10 ng/kg per minute to suppress the progression of angiostenosis. We performed vascular echocardiography twice a week and confirmed that there was no progression of angiostenosis following initiation of lipo-PGE1. We terminated infusion of lipo-PGE1 on day 36 (Fig. 3) and performed cardiac catheterization on day 45. His right renal artery was not visualized by angiography, and his right kidney was fed by collateral arteries, while vascular echocardiography revealed patency of his right renal artery with severe stenosis. We also confirmed celiac artery occlusion and superior mesenteric artery stenosis to be the same as those observed in previous echocardiography findings (Fig. 2b). His cardiac function gradually improved (Fig. 3). After his C-reactive protein levels returned to normal, 1 mg/kg per day of prednisolone was administered for 1 month, after which the dose was tapered every other 2 weeks. We observed him for 13 months after the termination of lipo-PGE1. The inflammatory findings remained negative and the diameters of abdominal aortic vessels were stable.

## Discussion

In this case report, we presented a Japanese 11-month-old baby boy with heart failure and TA and attempted to use lipo-PGE1 to prevent the progression of abdominal artery stenosis. To the best of our knowledge there is no report of the use of PGE1 in a baby or adult to prevent the progression of vascular stenosis, and this is probably the first reported case.

TA predominantly occurs in young women but is rarely reported in children. The incidence of newly diagnosed TA in children aged below 10 years was < 2% over the past 10 years in Japan [5]. As far as we know, only 11 cases of TA in babies aged < 1 year have been reported [6, 7].

Hypertension, bruit, and headache have been reported as common symptoms in adult- onset TA. In addition, aortic regurgitation and heart failure caused by coronary heart disease have also been reported in adults [8].

The most common symptoms were hypertension, fever, and vomiting in childhood-onset TA. Heart failure not related to aortic regurgitation or coronary involvement was rare in other cases involving children with TA [9, 10]. In the current case, two mechanisms were considered as the cause of heart failure: (1) afterload mismatch by hypertension and (2) myocarditis caused by TA. We speculated that it had been several months at initial presentation since the onset of TA because of the presence of cardiomegaly. The onset of TA may have occurred in this patient at the age of 7 months when he had a fever of unknown origin. Recently, childhood-onset TA has been gradually recognized. However, compared with the adult population, delayed diagnosis in children still occurs due to clinical manifestations being varied and nonspecific [11]. We suggest that heart failure should be considered a diagnostic feature of childhood-onset TA.

In the current case, we treated the patient with prednisolone as standard therapy; however, vascular stenosis progressed in spite of the treatment. Although cardiac catheterization was considered to be a treatment choice for vascular stenosis, we attempted the infusion of lipo-PGE1 owing to an increased risk of restenosis and vascular injury without complete abatement of inflammation [12].

Lipo-PGE1, launched in Japan in 1988, is a liposomal preparation containing PGE1 in lipid microspheres for gradual release. At the site of vascular lesions and inflammation, vascular endothelium gap spreads and phagocytosis of vascular endothelial cells becomes activated. As a result, vascular permeability for lipid microspheres increases. Therefore, an enhanced permeability and retention effect by lipo-PGE1 allows PGE1 to accumulate in the vascular lesion more effectively [13, 14]. In addition, lipo-PGE1 administered via a continuous intravenous drip is used to prevent patent ductus arteriosus for arterial duct-dependent heart disease or via a single intravenously administered injection for improving the superior mesenteric artery. On this basis, we believed that the progression of stenosis could be suppressed using a continuous intravenous drip of the drug that effectively accumulates at the site of inflammation. After treatment, no progression of stenosis was observed by abdominal echocardiography. No side effects were associated with the lipo-PGE1 treatment. Although this is only one case report, further cases should be analyzed. The present case suggested that lipo-PGE1 is effective in suppressing the progression of vascular stenosis associated with child TA.

## Conclusions

We conclude that TA is a rare disease; however, a differential diagnosis of heart failure with high blood pressure should be considered in babies. The progression of vascular stenosis may be suppressed by lipo-PGE1.

## Abbreviations

lipo-PGE1: Lipo-prostaglandin E1
LV: Left ventricular
PGE1: Prostaglandin E1
TA: Takayasu’s arteritis
